# Supplementary figures and images for: The Hippo kinase cascade regulates a contractile cell behavior and cell density in a close unicellular relative of animals
Source: eLife. 2024 Mar 22;12:RP90818. doi: 10.7554/eLife.90818 (PMC10959527; doi:10.7554/eLife.90818)

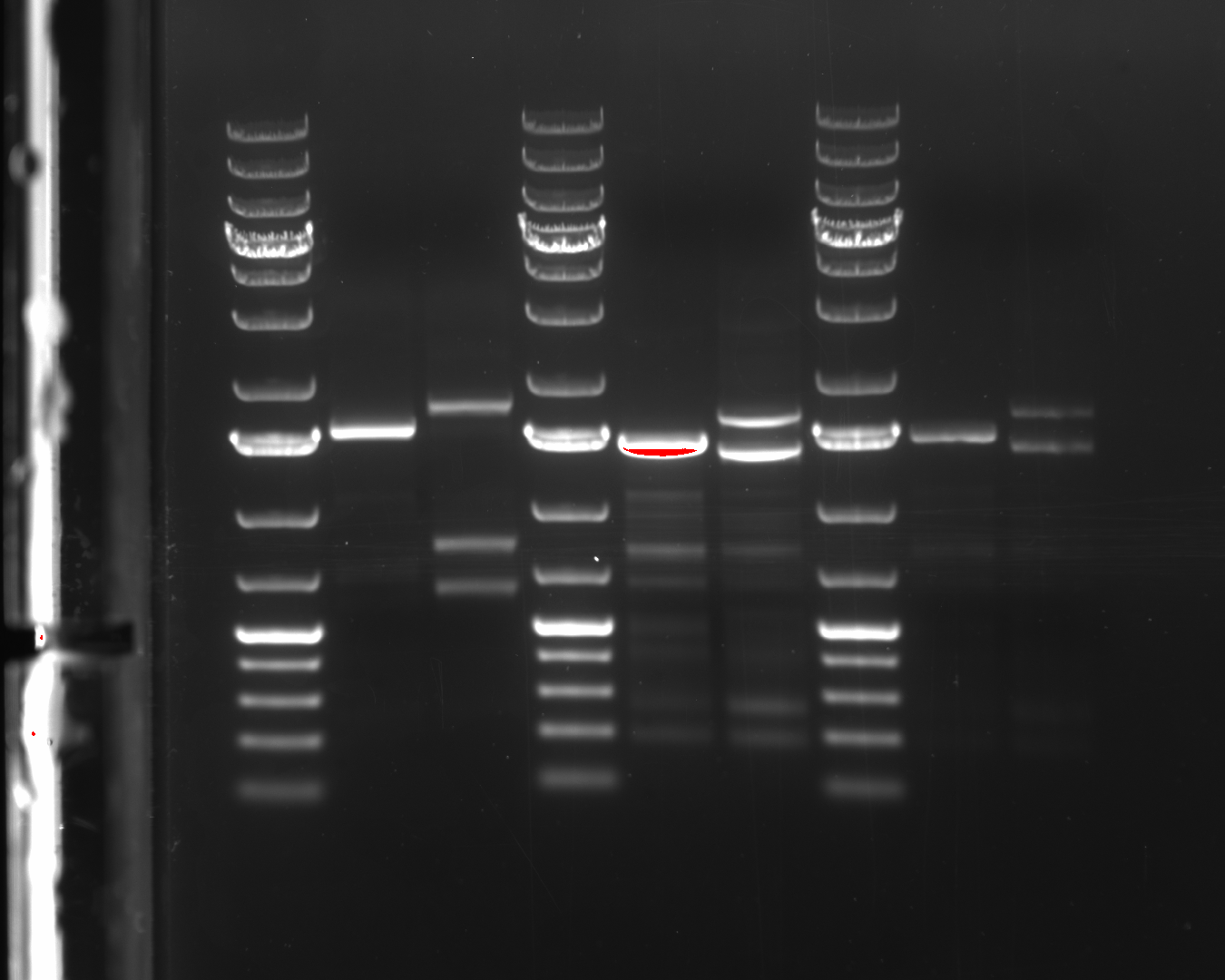

Supplement: Figure 1—figure supplement 1—source data 1. [file elife-90818-fig1-figsupp1-data1.tif]

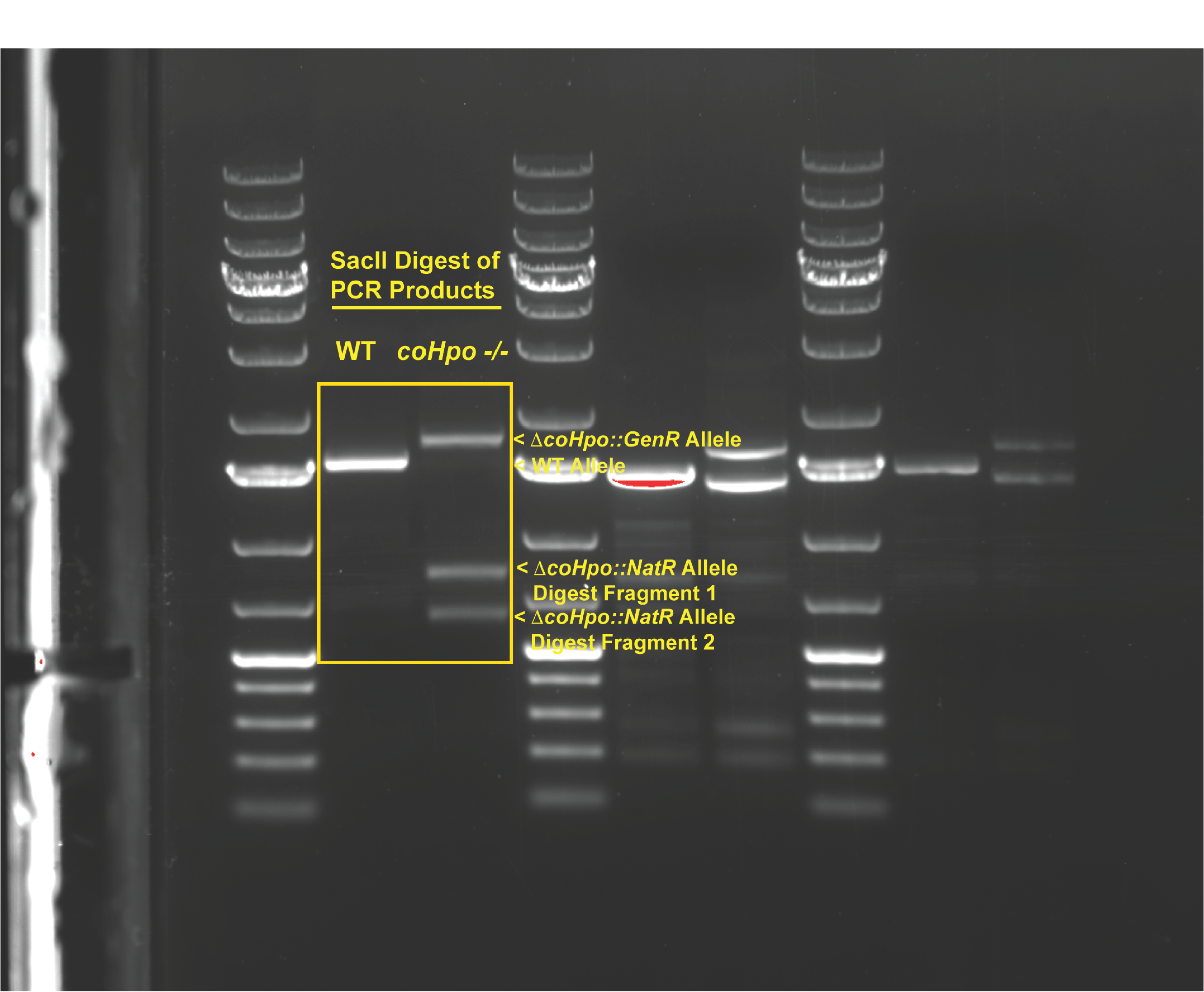

Supplement: Figure 1—figure supplement 1—source data 2. [file elife-90818-fig1-figsupp1-data2.tif]

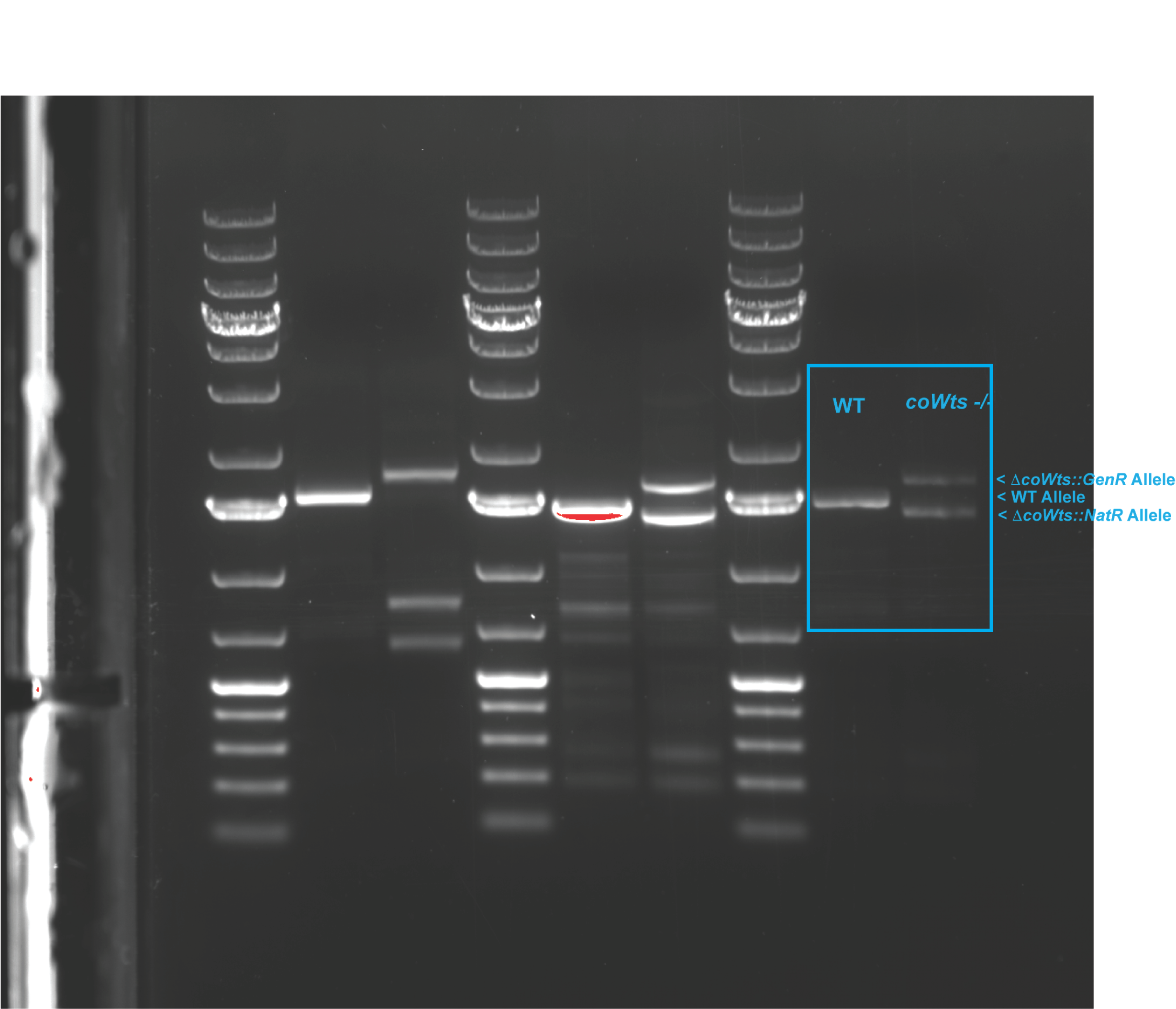

Supplement: Figure 1—figure supplement 1—source data 4. [file elife-90818-fig1-figsupp1-data4.tif]
